# From manual entry to machine precision: challenges and evolution of metadata schema development in collaborative research centers

**DOI:** 10.1186/s13104-026-07937-w

**Published:** 2026-07-06

**Authors:** Felix Engel, Claudia Giuliani, Manuel Watter, Aref Kalantari, Karin Schuller, Harald Binder, Klaus Kaier

**Affiliations:** 1 Institute of Medical Biometry and Statistics, Medical Faculty and Medical Center, University of Freiburg, Freiburg, Germany; 2 Helmholtz München, München, Germany; 3 Center for Integrative Biological Signaling Studies (CIBSS), University of Freiburg, Freiburg, Germany

**Keywords:** Research data management, Metadata schema development, Biomedical consortia, Ontology mapping, LLM-supported annotation

## Abstract

**Objective:**

Metadata standardization in collaborative biomedical research must balance interoperability with domain-specific detail. We describe a parent-template approach in which a baseline schema from the nephrology-focused CRC 1453 NephGen was adapted for the tumor-immunology CRC OncoEscape and the perinatal-immunology CRC Pilot.

**Results:**

The derivation process produced three structurally compatible yet vocabulary-divergent schemas. Pilot required the highest granularity (324 levels), followed by NephGen (287) and OncoEscape (283). Vocabulary reuse from the NephGen baseline was limited: 134 of 283 OncoEscape levels (47%) and 113 of 324 Pilot levels (35%) were retained unchanged. The main adaptations were not only expanded level lists, such as cell lines and mouse lines, but also new CRC-specific query dimensions, including “Oncogenes” in OncoEscape and “Timeline” in Pilot. In the context of AI-assisted extraction, we use the term *instruction set* to denote a schema that specifies target fields, expected granularity, example values, and validation resources for each metadata dimension, rather than a simple drop-down form or a free-text prompt template.

**Supplementary Information:**

The online version contains supplementary material available at 10.1186/s13104-026-07937-w.

## Introduction

The massive growth of digital information, often called a “data deluge,” turned research data management (RDM) from a nice-to-have into a core requirement for modern science [1–3]. Most management strategies rely on the FAIR principles, ensuring that digital research objects remain Findable, Accessible, Interoperable, and Reusable for humans and machines alike [2, 4]. Achieving these goals depends heavily on the creation of standardized, high-quality metadata that provides the necessary context for interpreting and reusing (raw) research data [1, 4, 5]. While being important, metadata documentation has been a labor-intensive and expensive process that may compete with other pressing research demands, often leading to the reality of “empty archives” where valuable data remains inaccessible [1, 6, 7].

To address this “metadata bottleneck,” various large-scale initiatives, such as the Collaborative Research Centers (CRCs), have pioneered bottom-up approaches to metadata schema development [1, 8]. These strategies focus on empowering scientists to define minimal, domain-specific schemas that are iteratively aligned with established standards by data stewards [9]. The present study follows this iterative process by adapting a conceptual baseline schema from the CRC 1453 “NephroGenetics” (NephGen) to meet the diverse data management needs of the CRCs “OncoEscape” and “Pilot”. By utilizing a parent template approach and implementing CRC-specific requirements as level refinements, this method balances cross-consortium standardization with the high granularity required for capturing specialized biomedical data regarding disease models, cell lines, and readouts.

This study focuses on biomedical CRCs in nephrology, tumor immunology, and perinatal immune development. The resulting schemas were not intended to replace assay-specific reporting standards such as MIAME, MIGS/MIMS, or ISA-Tab, nor generic administrative standards such as Dublin Core or DataCite. Instead, they operate at a different layer: a CRC-wide discovery and reuse layer that must cover heterogeneous experimental designs within one shared search environment. We therefore reused generic administrative concepts where possible and mapped domain concepts to established ontologies such as EFO, BRENDA Tissue Ontology, and Cellosaurus, while retaining a custom parent-template backbone for CRC-specific retrieval dimensions such as developmental stage, oncogenic perturbation, mouse line, and readout. The contribution of the parent-template approach thus lies in combining a stable structural backbone with curated local vocabularies rather than in competing with community reporting standards. This manuscript should therefore be read as the architectural blueprint of the schemas, whereas our companion papers evaluate downstream LLM-based annotation performance using these structures.

## Methods

**Fig. 1:**
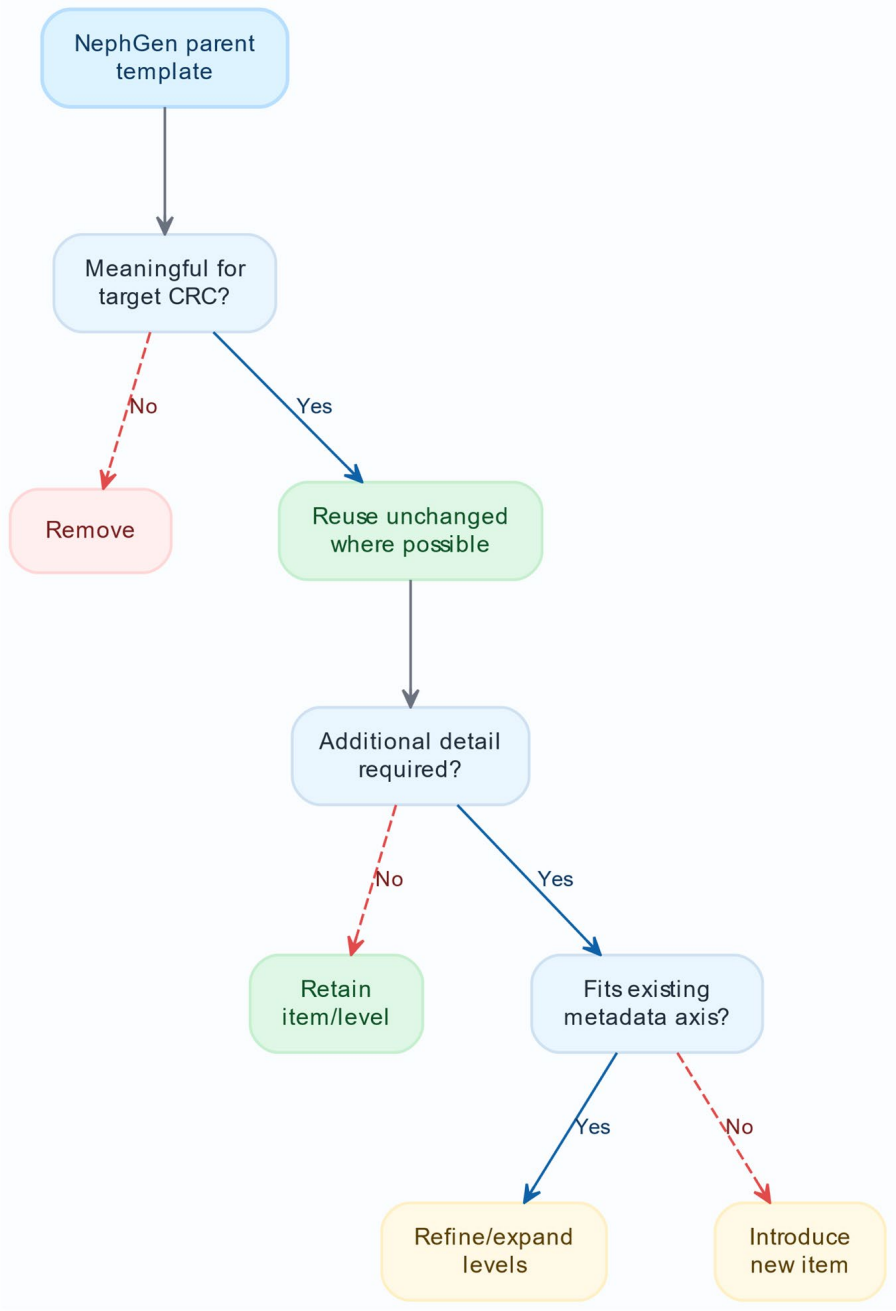
Decision workflow for deriving CRC-specific schemas from the NephGen parent template

The development of the metadata schemas followed an iterative, bottom-up approach designed to balance standardization with domain-specific flexibility. The schema initially developed for the NephGen served as the conceptual baseline [10]. This schema was designed to capture high-dimensional data regarding kidney diseases and gene regulation. To address the data management needs of two subsequent consortia, OncoEscape and Pilot, we utilized the NephGen schema as a parent template (see Fig. 1). The adaptation process involved stripping the baseline schema of consortium-specific terminologies while retaining the structural backbone, which includes modules for publication metadata, data location, experimental classification and accessibility information (e.g., storage formatting and access rights).

Schema derivation followed a gap-analysis workflow. Starting from NephGen, we first removed levels that were clearly kidney-specific and not meaningful in the target CRC. We then asked for each missing requirement whether it could be expressed within an existing metadata axis or whether it represented an additional retrieval dimension. Shared conceptual axes were implemented as level refinements within existing items; genuinely new retrieval dimensions were modeled as new items. For example, expanded tumor-related terms were introduced as curated levels, whereas OncoEscape received the dedicated item Oncogenes because oncogenic drivers constitute an explicit search dimension in that consortium. Pilot, in turn, received the item “Timeline” because developmental stage cuts across organism, tissue source, and intervention and could not be represented unambiguously as a subtype of Health Status without conflating disease state and developmental time. Requirements were derived from the CRC proposals and iteratively reviewed with researchers, PIs, and local data stewards. Each CRC went through approximately two to three iterative review rounds. Feedback was collected in internal schema-review meetings with PIs and data stewards and in email-based revisions, and typically resulted in added or removed levels, renamed labels, or clarified item scope rather than changes to the structural backbone. These consultations were intended to establish practical coverage and comprehensibility, not to constitute a formal consensus study. These consultations were intended to establish practical coverage and comprehensibility, not to constitute a formal consensus study.

The schemas were implemented as JSON-based configurations within the *fredato* research data management tool, as described previously [9]. Data items and levels were mapped to established ontologies (e.g., Experimental Factor Ontology EFO, BRENDA Tissue Ontology, Cellosaurus) where possible. While generic administrative items and standard tissue sources were highly mappable to these standard ontologies, many of the highly specific experimental perturbations or custom, locally established mouse lines remained as unmapped, CRC-specific entities. The distinct items and levels for each schema were curated in spreadsheet formats to allow for easy editing before being converted into the final machine-readable JSON format. This setup enables researchers to annotate datasets via a web-based interface that dynamically presents the specific items and levels relevant to their consortium’s research focus.

During schema development, candidate versions were tested exploratively on representative CRC publications and datasets to identify missing items, ambiguous labels, and over- or under-granular level lists. These tests informed iterative revisions, but they were not prospectively logged and are therefore not reported as a formal validation experiment. To make the manuscript self-contained, we instead summarize the structured downstream applications in which the final schemas were used for LLM-supported annotation. In OncoEscape, a 4-step GPT-4o/PubTator workflow was applied to 23 articles and produced 604 annotation suggestions, corresponding to a mean of 26.3 biomedical entities per article, including 19.6 schema-related and 6.7 PubTator-validated entities. Expert validation in face-to-face interviews yielded an overall precision of 98% (95% CI 94%-100%); schema-related entities alone reached 97% precision (95% CI 91%-100%), and PubTator-validated entities reached 100% precision [11]. In NephGen, a grounded multi-model workflow was evaluated on six articles after initial feasibility testing of 14 LLMs, of which eight reliably completed the annotation workflow. Across models, overall precision was 91.3% (95% CI 87.4%-94.6%); the best-performing models reached 95.7% for GPT-4.1, 97.7% for GPT-4o Mini, and 93.6% for Gemini 2.0 Flash [8]. In Pilot, a three-step search-augmented workflow was used to identify and annotate datasets across six articles and multiple public repositories. The workflow identified all datasets referenced in the articles and generated schema-compliant dataset-level annotations; Gemini 2.5 Pro achieved the highest precision (97.1%, 95% CI 94.9%-98.9%), while repository-only extraction was more precise than repository-plus-article extraction across all models (91.9% vs. 88.3%, *p* = 0.004) [6]. These studies do not validate the derivation procedure itself, but they show that the resulting schemas were operationally usable as structured guidance for downstream metadata annotation. The Pilot and OncoEscape schema configurations described in this study are publicly available in the FreiData repository, including mappings on established ontologies and organizing principles for knowledge graphs [12, 13].

Overlap between schemas was calculated conservatively as exact identity of curated level labels in the final schemas. Near-synonyms or closely related variants were not collapsed. For example, labels such as HEK293 and HEK293T were treated as distinct levels because the aim of the comparison was to quantify directly reusable curated vocabulary, not semantic similarity after ontology normalization.

## Results

The derivation process resulted in three distinct yet structurally compatible schemas. While core metadata items concerning data provenance and file location remained constant, classification items and their levels varied substantially according to the scientific focus of each CRC (Table 1).

**Table 1:** Items and number of levels of the three schemas

Nephgen schema	OncoEscape schema	Pilot schema
Organism (9)	Organism (3)	Organism (4)
		Timeline (3)
Cell Lines (14)	Cell Lines (68)	Cell Lines (101)
Tissue Source (46)	Tissue Source (21)	Tissue Source (34)
	Interventions (3)	Interventions (31)
Health Status (8)	Health Status (1)	
Mouse Line (12)	Mouse Line (62)	Mouse Line (46)
Sample Preparation (52)	Sample Preparation (52)	Sample Preparation (52)
	Oncogenes (17)	
Sample Processing (6)	Sample Processing (6)	Sample Processing (6)
Readout (50)	Readout (50)	Readout (47)
Probes (90)		
Total Levels: 287	**Total Levels: 283**	**Total Levels: 324**

Table 1 and Supplement 1 jointly show that differences occur in two ways: new items introduced for CRC-specific query dimensions, and redistribution of detail across existing items.

Pilot contained 324 curated levels, NephGen 287, and OncoEscape 283. Quantitative reuse of the NephGen vocabulary was limited: 134 levels were retained unchanged in OncoEscape, corresponding to 47% of the final OncoEscape vocabulary, and 113 levels were retained unchanged in Pilot, corresponding to 35% of the final Pilot vocabulary. These differences reflected both new CRC-specific query dimensions and redistribution of granularity across existing items, including expanded cell-line and mouse-line vocabularies, the `Oncogenes` item in OncoEscape, and the `Timeline` item in Pilot.

While all schemas capture information about the item “Organism”, the Pilot schema introduced a unique “Timeline” item with levels such as *embryonic/prenatal*, *perinatal–weaning*, and *adult*. This addition addresses the specific requirement of TRR 359 to document the temporal dynamics of immune development, a factor less critical in the static disease models of the other CRCs. Consequently, the Pilot schema moves away from the static ‘Health Status’ item used by NephGen (which lists specific kidney diseases), relying instead on this developmental staging to define the sample context.

The NephGen schema exhibits the highest granularity in the “Tissue Source” item (46 levels), specifically enumerating renal substructures (e.g., *juxtaglomerular apparatus*, *proximal tubule*). This aligns with CRC 1453’s focus on dissecting the genetic basis of kidney function at the substructure level. In contrast, the OncoEscape and Pilot schemas cut back on tissue granularity (21 and 34 levels) to make room for expanded “Cell Line” vocabularies. The Pilot schema includes 101 cell line levels to cover diverse immune cell subsets. This massive expansion was a direct result of early explorative feedback, where Pilot researchers noted that standard tissue classifications failed to capture the highly specific immune cell populations central to their datasets. OncoEscape, with 68 levels, focuses specifically on the cancer cell lines (like HeLa or K562) needed to study tumor-immune interactions. In addition, OncoEscape and Pilot list significantly more “Mouse Lines” (62 and 46 levels, respectively) than NephGen (12 levels), reflecting the necessity to annotate specific genetic alterations and knock-outs in mouse models.

To address the central hypothesis of CRC 1479, that signaling pathways drive immune evasion, the OncoEscape schema introduced a dedicated “Oncogenes” item (17 levels), listing specific drivers such as *KRAS-G12D*, *BRAF-V600E*, and *MYC*. This item is absent in the other schemas. In contrast, the NephGen schema retains a unique “Probes” item with 90 levels, reflecting the consortium’s heavy reliance on specific antibodies and primers for characterizing renal tissue architecture.

At the item level, some methodological categories remained comparatively stable across schemas, particularly `Sample Preparation` (52 levels in each schema) and `Readout` methods (47–50 levels). However, specific modifications were made where necessary; for example, the Pilot schema includes specific “Interventions” (31 levels) tailored to immunological challenges (e.g., *maternal inflammation*, *poly I: C injection*), whereas OncoEscape emphasizes genetic and pharmacological perturbations relevant to tumor suppression.

## Discussion

### Empirical schema-design lessons

The comparison of the three schemas presented in this study illustrates a fundamental challenge in research data management: the trade-off between schema completeness and practical usability. Our empirical comparison supports three core design lessons for CRC-wide schemas. First, the structural backbone (e.g., publication metadata, data location) is highly portable across different biomedical disciplines. Second, the curated vocabulary requires substantial domain-specific adaptation, as evidenced by the low quantitative reuse of the NephGen baseline vocabulary in OncoEscape (47%) and Pilot (35%). Third, granularity should follow the specific retrieval logic of each consortium rather than being maximized globally. New items, such as “Timeline” or “Oncogenes”, should only be introduced when they represent a genuinely new retrieval dimension that cannot be expressed within existing items without conflating concepts. While these empirical lessons optimize schemas for human curators, we argue that the entire field of schema development is currently at a crossroads.

### Outlook: AI-supported metadata workflows

The following sections should be read as a perspective on possible future uses of the schema structures described above. The present study did not evaluate dynamic schemas or agentic LLM workflows directly; these topics are discussed as implications for future research and implementation.

So far, the design of our schemas was constrained by the effort scientists were able to invest into manual annotation [14–18]. To ensure researcher participation, schemas had to be concise, with a limited granularity of data items such as “Sample Preparation” or “Readout” presented as manageable drop-down lists. The introduction of Large Language Models (LLMs) is currently changing these requirements [19]. Our recent work indicates that LLMs may support the automated extraction of complex metadata fields from full texts with promising precision [6, 8]. This means that future data schemas will have to address other agents — in addition to or instead of human annotators. The challenge is to achieve a wider scope of metadata and higher precision, a potential benefit of all automation [20]. Consequently, schemas could evolve from minimized query templates into extensive ‘instruction sets’ for agentic workflows [21]. For example, an AI-oriented instruction set for the item *Cell Lines* would not only list accepted values, but also specify the expected granularity, provide example values such as HeLa, K562, or HEK293T, indicate whether primary immune cell subsets should be captured separately, and link candidate annotations to validation resources such as Cellosaurus. In this form, the schema serves as a structured prompt that conveys field, granularity, examples, and a grounding resource in a single specification. In this model, the AI does not only extract text but may also validate decisions against schema constraints and retrieve external context from the web [22]. Such workflows could enable a depth of metadata capture that would be unfeasible in a purely manual workflow [23].

AI does not just emerge as an agent in metadata generation but also in aggregation and retrieval. Our schemas were developed with queries by scientists, both from within the consortia and without, in mind. These target groups are now complemented with a range of AI-supported applications retrieving information for various purposes. While the previous schema approach was to reduce the complexity of available information to a level that could be handled by humans, research metadata now need to be made AI-ready [24]. This no longer implies reduction of complexity but rather reduction of ambiguity. The focus of future AI-based applications might not lie with metadata but with the research data themselves. Metadata will be needed to contextualize these and ensure correct interpretation. For now, schema design will have to address several target groups as scientists can be expected to continue querying databases directly.

### Balancing uniformity with dataset-specificity

The presented schemas were designed as uniform standards for their respective CRCs, reflecting the shared scientific consensus of the participating groups. However, as shown by the distinct item sets in the Pilot and OncoEscape schemas, a “one-size-fits-all” approach inevitably creates friction when individual datasets deviate from the consortium’s primary focus, as observed in a schema-guided annotation study using the OncoEscape schema [11]. Rigid uniformity can lead to the misclassification of edge cases or the loss of specific details that do not fit predefined categories. LLM-based approaches offer a pathway to resolve this tension by enabling dynamic schema adaptation. Instead of forcing all datasets into a static structure, future workflows could employ flexible schemas [25]. Here, the LLM aligns the core metadata with the CRC standard but adapts specific fields to reflect the evolving research focus of the consortium. This prevents the schema from becoming a rigid cage while avoiding the chaos of uncontrolled growth. It ensures that the metadata structure reflects the dimensions along which researchers organize their thoughts across a series of publications, rather than reacting impulsively to a single dataset. The core administrative data stays standard, but the scientific descriptions can flex to fit the actual research output.

### From closed vocabularies to grounded open annotation

Our results show just how much effort it takes to curate controlled vocabularies, like the 101 cell lines in the Pilot schema. Keeping lists like that up to date is work-intensive and they inevitably lag behind the actual research. A shift towards more open annotation strategies appears viable, provided that free additions are scientifically validated [26]. Recent work on biomedical entity resources and LLM-assisted biocuration supports this hybrid approach, particularly when generated annotations are linked to external validation resources and evidence sources [27–29]. However, any shift toward LLM-assisted annotation in biomedical contexts must address well-documented challenges including reliability, bias, privacy, and the risk of error propagation [20, 23]. Such workflows therefore require schema guidance, audit trails, and continued human oversight rather than unconstrained generation [26, 30]. In this setting, the role of data stewards shifts rather than disappears: from maintaining exhaustive drop-down lists toward curating the semantic backbone, auditing model suggestions, and governing schema evolution over time [30]. Grounding LLM-generated annotations in established repositories such as PubTator 3.0, or using frameworks such as BioChatter, could help verify the existence of entities (e.g., a specific gene or cell line) without pre-populating them in the schema [8, 27–29]. In this scenario, the schema acts as a semantic backbone. It defines *what* to look for, but it also provides crucial context on granularity. For instance, the presence of broad terms like ‘brain’ or ‘liver’ signals to the AI that it shouldn’t drill down to specific cell types unless necessary. Effectively, the existing controlled vocabulary serves as a set of ‘few-shot’ examples, guiding the LLM on the expected level of detail [31]. To prevent these lists from degenerating into a mess of synonyms or unrelated entries, we will likely need a strategy for pruning and validation—finding a robust ruleset that the AI can follow to maintain high data quality over time [30]. This reduces the maintenance burden of the schema while preserving the reliability of controlled vocabularies.

### Implications for technical implementation and discovery

The transition from rigid to flexible, LLM-driven schemas presents challenges for existing technical infrastructures. The current implementation of these schemas in the *fredato* system relies on fixed JSON structures to facilitate indexing and internal search [9]. Moving toward open or dynamic schemas complicates this architecture, as traditional database queries struggle with variable fields. However, the same AI technologies driving annotation can also enhance retrieval. If metadata generation becomes more fluid, search mechanisms must evolve from keyword matching within fixed fields to semantic search capabilities that can interpret the context of unstructured or semi-structured metadata [8]. Therefore, while the schemas presented here provide a necessary foundation for current data management, they should be viewed as a transitional step toward a more fluid, AI-integrated ecosystem where discovery is driven by content relevance rather than rigid schema compliance.

### Limitations

The primary limitation of the work presented here lies in the original intent of the schemas. They were fundamentally designed for manual annotation and information retrieval, necessitating a reduction in complexity to support researcher participation and practical usability. Consequently, their application in the emerging LLM-based workflows described in the discussion represents a repurposing of legacy structures. These schemas impose a major constraint: Limiting the “search space” of the AI to what was previously manageable for humans, rather than leveraging the full semantic potential of large language models. This introduces the risk of ‘anchoring bias’, whereby an AI that is artificially constrained by a legacy drop-down list may fail to capture complex biological nuances present in the raw data, simply because the appropriate descriptive term is not present in the restricted schema. There is also a mismatch between how fast AI is moving and how slow schema governance tends to be. LLM capabilities are evolving quickly, but the consensus-driven process of building a schema is still slow and rigid. These points suggest that the CRC schemas alone might not be the optimal grounding for LLM-based metadata annotation. More complex resources like ontologies or knowledge graphs might be more appropriate. However, the high precision rates for LLM-based data annotation reported in our previous research might, in fact, be largely caused by the strong restriction from limited schemas. Future work must address how to liberate metadata definition from manual-era constraints without losing the semantic interoperability that schemas provide.

Theoretically, automated extraction allows for limitless granularity, leading to fully dynamic, high-dimensional metadata environments. But it remains to be seen if this information will actually be used and how. Traditionally, metadata were structured to be conveniently queried. This advantage would be lost if their structure becomes too complicated or if they were to become unstructured, after all. On the other hand, LLMs are not just employed in metadata production but also in data retrieval and might be able to process even complex metadata structures. Then again, LLM-based agents might not rely on larger quantities of traditional metadata but require other kinds of information to evaluate the evidence directly.

So far, all AI-supported process pipelines that we operate have been specifically designed to keep human specialists in the loop. Departing from simplified metadata schemas in favor of less restricted groundings of LLM processes might soon overexert scientists. Improvement of models might, eventually, render human interaction unnecessary but such a development cannot be predicted with certainty. For the time being, liberation of LLM predictions will need to stay within the realm of what humans can handle.

Finally, evaluating a metadata schema at the time of its creation is inherently challenging. While the CRC schemas are designed for long-term data reusability and cross-consortium integration, our validation was limited to their operational usability in downstream annotation pipelines. A formal, short-term validation against a narrow set of initial use cases is difficult to implement without introducing bias, and it may not fully capture the schema’s future utility or structural robustness over a longer funding period.

## Supplementary Information

Below is the link to the electronic supplementary material.

Supplementary Material 1.

## Data Availability

The datasets used and/or analysed during the current study are available from the corresponding author on reasonable request.
