# Supplementary material for: From manual entry to machine precision: challenges and evolution of metadata schema development in collaborative research centers
Source: BMC Res Notes. 2026 Jul 6;19:277. doi: 10.1186/s13104-026-07937-w (PMC13339696; doi:10.1186/s13104-026-07937-w)
Supplement: Supplementary file 1 — Supplementary Material 1. [file 13104_2026_7937_MOESM1_ESM.pdf]

Schema comparison: Nephgen vs OncoEscape vs Pilot

Columns: Nephgen-Schema (left), OncoEscape-Schema (middle), Pilot-Schema (right). Identical levels are aligned side by side within each section. Blank cells indicate levels only present in the other schema(s).

Nephgen-Schema	OncoEscape-Schema	Pilot-Schema
**Organism**	**Organism**	**Organism**
• Cell line	• Cell line	• Cell line
• Human	• Human	• Human
• Mouse	• Mouse	• Mouse
• C. elegans		
• Rabbit		
• Rat		
• Fruit Fly		
• Zebrafish		
• Xenopus		
		• Pig
		**Timeline**
		• embryonic / prenatal
		• perinatal – weaning
		• adult
**Cell Lines**	**Cell Lines**	**Cell Lines**
293 (HEK 293)		HEK293
293A (HEK 293A)		
293T (HEK 293T)	HEK293T	HEK293T
AC16 Human Cardiomyocyte Cell Line		
HAF		
HeLa		HeLa
HeLa 229		
HL-1		
HUVEC		HUVEC
THP-1	THP-1	THP-1
MDCK		MDCK
mIMCD-3		
LLC-PK1		
RPTEC		

	B16.F10	
	B16.F10luc/GFP	
	B16.F10OVA	
	AT-3 OVA	
	RENCA	
	KP1.9	
	4434-BRAFV600E	
	MC38	
	MC38 OVA	
	MC38ROR1+/GFP+/Luc+	
	RMB1	
	C1498	
	C1498GFP+ luc+	
	Yumm1.7	
	Yumm1.7 OVA	
	MODE-K	
	JIMT-1 breast cancer	
	32D	
	Platinum E	
	WEHI-3B	
	WEHI-3BLuc/GFP	
	WEHI-3BCD155KO	
	MOLM-13	
	BAF-3	
	MV4-11	
	MV4-11Luc+	
	MV4-11NT Luc+	
	MV4-11JunD KO (G4) Luc+	
	MV4-11cJun KO (G11) Luc+	
	MV4-11JunD KO (G64) Luc+	
	MV4-11cJun KO (G69) Luc+	
	MV4-11AXL KO (G1) Luc+	
	OCI-AML2GFP+Luc+	
	OCI-AML3	
	OCI-AML3GFP+Luc+	
	Kasumi-1	
	HL-60	HL-60
	SEM	
	E2a-PBX	

	A20GFP+Luc+	
	OCI-AML3hp53 1961 (p53 KD) YFP+ dsRed+	
	OCI-AML3hp53 Renilla (p53 WT) YFP+ dsRed+	
	RP1199.6	
	RP1199.1	
	RP1201.1	
	RP1209.1	
	RP12086	
	PancROR1+/GFP+/Luc+	
	STC-1	
	NB-4	NB-4
	ML-2	
	K562	K562
	KG-1	KG-1
	KG-1α	KG-1a
	MUTZ-8	
	BV2	
	FL83B	
	HLE	
	Colo800	
	murine embryonic fibroblasts	MEF (C57BL/6) cell
	OV-90	
	BxPC3	BxPC-3
	NCI-H2405	
	Steinberger NFAT-reporter	
	SMMC-7721	
	HepG2	HEPG2
		3T3 cell
		A2780
		A549
		AGS
		AsPC-1
		BEAS-2B
		BHK-21
		BT-474
		C2C12
		C6
		Caco-2 cell
		Calu-3

		Calu-6
		Capan-1
		Capan-2
		CCRF-CEM
		CHO cells
		CHO-K1
		COS-7
		Daoy
		DLD-1 cell
		DU145
		EA.hy926
		F9
		H1
		H1299
		H1975
		H460
		H9
		HaCaT
		HCT 116 cell
		HEP-2
		Hep3B
		HOS
		HT-29
		Huh7
		HUV-EC-C
		J774A.1 cell
		Jurkat
		Jurkat J6
		L929
		LN229
		LNCaP clone FGC
		LoVo
		MCF10A
		MCF7 cell
		MDA-MB-231
		MDA-MB-453
		MDA-MB-468
		MG-63
		MIA PaCa-2

		MKN28
		MKN-45
		MOLT-4
		MRC-5
		NCI-H1299
		NCI-H1975
		NCI-H460
		NIH-3T3
		NT2-D1
		NTERA-2 cl.D1
		OVCAR-3
		OVCAR-4
		P19
		PANC-1
		PC-3
		PLC/PRF/5
		Raji
		Ramos
		RAW 264.7 cell
		RWPE-1
		Saos-2
		SH-SY5Y
		SK-BR-3
		SK-N-SH
		SK-OV-3
		SupT1
		SW480
		SW620
		T47D
		T98G
		U251
		U2OS
		U87MG
		U937
		Vero
		WI-38
**Tissue source**	**Tissue Source**	**Tissue Source**
• Adrenal gland	• Adrenal gland	

• **Blood**	• **Blood**	• **Blood**
• Blood plasma	• Blood plasma	• Blood plasma
• Blood serum	• Blood serum	• Blood serum
• Whole blood	• Whole blood	• Whole blood
		• Blood cells
• Bone marrow	• Bone marrow	• Bone marrow
• Brain	• Brain	
• Embryonal tissue	• Embryonal tissue	• Embryonal tissue
• Heart	• Heart	• Heart
• Intestine	• Intestine	• Intestine
**Kidney**	• Kidney	• Kidney
• Head kidney		
**Juxtaglomerular apparatus**		
• Extraglomerular mesangial cell		
• Juxtaglomerular cell		
• Macula densa		
• Kidney cancer cell		
• Nephridium		
**Nephron**		
• Renal corpuscle		
**Renal tubule**		
• Collecting duct		
• Henles loop		
• Renal clear cell		
• Renal distal tubule		
• Renal proximal tubule		
• Renal tubule epithelium		
• Opisthonephros		
• Renal artery		
• Renal cortex		
• Renal epithelium		
• Renal medulla		
• Renal neoplasm cell		
• Renal parenchyma		
• Renal pyramid		
• Trunk kidney		
• Tubulointerstitium		
• Liver	• Liver	• Liver
• Lung	• Lung	• Lung
• Lymph node	• Lymph node	• Lymph node

• Nerve	• Nerve	• Nerve
• Skin	• Skin	• **Skin**
		• Dermis
		• Epidermis
		• Fascia
• Spleen	• Spleen	• Spleen
• Thymus	• Thymus	• Thymus
• Urine	• Urine	
	• Feces	
• Vascular system	• Vascular system	• **Vascular system**
		• Abdominal aorta
		• Aortic arch
		• Aortic root
		• Inferior vena cava
		• Superior vena cava
		• Adipose tissue
		• **CNS**
		• Brain
		• Spinal cord
		• Dura
		• Leptomeninges
		• Small Intestine
		• Colon
	**Interventions**	**Interventions**
	• **Genetic modification**	• **Genetic modification**
	**Genes of interest**	**Genes of interest**
		• TGFB1
		• MRC1
		• MYD88
		• GP91
		• P47
		• HIF1A
		• NOS2
	• **Pharmacological treatment**	• **Pharmacological treatment**
	**Pharmacological/dietary interventions**	**Pharmacological/dietary interventions**
		• High fat diet
		• Tamoxifen induction
		• DSS treatment
		• Csf1R inhibitor treatment

		• Diphteria toxin treatment
		• Antibiotics
		• **Infection**
		• Bacille Calmette Guérin (BCG)
		• Guillain-Barre syndrome (GBS)
		• Staphylococcus aureus
		• Cytomegalovirus
		• Mycobacterium marinum
		• Mycobacterium avium
		• Legionella pneumophila
		• **Injection**
		• Poly I:C
		• Maternal inflammation
		• CpG
		• R848
		• **Housing condition**
		• Individual housing
		• Germ-free
	• Surgical Intervention	
	Surgical Interventions	
**Health status**	**Health Status**	
• Autosomal Dominant Polycystic Kidney Disease (ADPKD)		
• Nephronophthisis		
• Chronic Kidney Disease (CKD)		
• Nephrotic syndrome		
• Clear cell renal carcinoma (ccRCC)		
• Von Hippel Lindau syndrome		
• Congenital abnormality of the kidney		
• Congenital abnormality of the ureter		
	• Cancer	
**Mouse line**	**Mouse Line**	**Mouse Line**
C57BL/6J (wildtype)	C57BL/6J (wildtype)	C57BL/6J (wildtype)
		C57BL/6J RccHsd (wildtype without nnt mutation)
Ksp-Cre		
Pax8-Cre (Pax8rtTA)		
Pgk-Cre		
Pod-Cre		

LC1-Cre		
Tam-Cre		
Pkd1-flox		
Pkd1-		
Dnajb11 tm1a		
Dnajb11-flox		
Dnajb11-		
	C57BL/6-Nrastm1Tyj/J X Vav-Cre	
	C57BL/6JCya-Tigitem1/Cya	
	Tigitfl/fl;CD4cre/+	
	CD155-/- mice	
	Tigitfl/fl;Zbtb46cre/+	
	Gal9-/-	
	Nlrp3-/-	
	Pycard-/-	
	Casp1-/-	
	Nt5e/Cd73-/-	
	Gsdmd-/-	
	Il1r1-/-	
	Cmtm6-/-	
	Osm-/-	
	Osmr-/-	
	FLT3-ITD	
	Ddit3-/-	
	Rag1tm1Mom	
	Rag2-/-gc-/-	
	Tet2-ko (B6(Cg)-Tet2tm1.2Rao/J	
	Apcflox/flox	
	Col7a1fl/fl	
	Pycard fl/fl	
	Xbp1 fl/fl	
	Adora2 fl/fl	
	Dnmt3afl/+	
	Osmr fl/fl	
	Il1r1 fl/fl	
	Trp53fl/fl	
	Trp53LSL-R175H	
	KrasLSL-G12D	
	Vhl fl/fl	

	Rb1 fl/fl	
	Tak1 fl/fl	
	Atf6 fl/fl	
	Ptpn11 D61Y/+	
	SCLtTA/TRE-Cre	
	SCL-Cre	
	Osterix-Cre	
	LepR-Cre	
	Prx1-Cre	
	CD4-Cre	
	CX3CR1 ERT2 Cre	Cx3cr1-CreER
	HexbCreERT2:R26RConfetti	
	Mrc1CreERT2:R26RConfetti	Mrc1-CreER
	Cxcr4CreERT2:R26RConfetti	Cxcr4-CreER
	Ksp1.3-CreERT2	
	Ksp1.3-CreERT2; Vhl fl/fl; Trp53 fl/fl; Rb1 fl/fl	
	Mx1 Cre	
	LysM Cre iDTR	LysM-Cre
	Cd4 Cre ERT2	
	Villin-CreERT2	Villin-Cre
	Rosa26::CreERT2	
	Tet2 fl/flMx1-Cre	
	Rosa26::Cre ERT2 KrasG12V	
	Rosa26::Cre ERT2 Jak2-V617F FLEX/+	
	nATF6liv	
	Cas9	
	MMTV-PyMT	
	ERAI	
	OT-1/Rag2-/-	OT-I
		beta-actin-dsRed
		beta-actin-GFP
		CAG-CreER
		Ccr2-/-
		Cd11c-Cre
		Cd45.1
		Clec4F-Cre
		Csf2rb-/-
		Csf2rb-flox
		Cx3cr1-Cre

		Cx3cr1-GFP
		DTA-flox
		DTR-flox
		gp91-/-
		Hif1a-flox
		Ifnar1-flox
		iNos-/-
		Irf8-/-
		Ms4a3-Cre
		MyD88-flox
		Nav1.8-Cre
		p47-/-
		Pdgfra-CreER
		Sftpc-CreER
		Tgfbr2-flox
		Tomato-flox
		YFP-flox
		Clec9a-cre x Rosa-tdTomato
		Clec9a-cre x Rosa-YFP
		Clec9a-cre x CD64-DTR
		RORgt-eGFP
		Cd11c-Cre x Stat1-flox
		Clec9a-cre x Stat1-flox
		Clec9a-cre x Irf8-flox
		OT-I x Nr4a1-eGFP
		OT-II (Thy1.1)
		Clec9a-cre x Irf4-flox-GFP
		Clec9a-cre x EP2-KO x EP4-flox
**Sample preparation**	**Sample Preparation**	**Sample preparation**
**Cultured cells**	**Cultured cells**	**Cultured cells**
• Adipocyte	• Adipocyte	• Adipocyte
• Cardiomyocyte	• Cardiomyocyte	
• Dendritic cell	• Dendritic cell	• Dendritic cell
• Embryonic cardiomyocytes	• Embryonic cardiomyocytes	• Embryonic cardiomyocytes
• Endothelial cell	• Endothelial cell	• Endothelial cell
• Epithelial cell	• Epithelial cell	• Epithelial cell
• Fibroblast	• Fibroblast	• Fibroblast
• hiPSC-CM	• hiPSC-CM	• hiPSC-CM
• hiPSC-FB	• hiPSC-FB	• hiPSC-FB

• Immune cell	• Immune cell	• Immune cell
• Leukocytes	• Leukocytes	• Leukocytes
• Lymphocytes	• Lymphocytes	• Lymphocytes
• Macrophage	• Macrophage	• Macrophage
• Monocytes	• Monocytes	• Monocytes
• Natural killer cells	• Natural killer cells	• Natural killer cells
• Neuronal cell	• Neuronal cell	• Neuronal cell
• Neurones	• Neurones	• Neurones
• Neutrophils	• Neutrophils	• Neutrophils
• Oocyte	• Oocyte	• Oocyte
• Pericytes	• Pericytes	• Pericytes
• Platelet	• Platelet	• Platelet
• Smooth muscle cells	• Smooth muscle cells	• Smooth muscle cells
• T cells	• T cells	• T cells
		• B cell
**Isolated cells**	**Isolated cells**	**Isolated cells**
• Adipocyte	• Adipocyte	• Adipocyte
• Cardiomyocyte	• Cardiomyocyte	
• Dendritic cell	• Dendritic cell	• Dendritic cell
• Embryonic cardiomyocytes	• Embryonic cardiomyocytes	• Embryonic cardiomyocytes
• Endothelial cell	• Endothelial cell	• Endothelial cell
• Epithelial cell	• Epithelial cell	• Epithelial cell
• Fibroblast	• Fibroblast	• Fibroblast
• hiPSC-CM	• hiPSC-CM	• hiPSC-CM
• hiPSC-FB	• hiPSC-FB	• hiPSC-FB
• Immune cell	• Immune cell	• Immune cell
• Leukocytes	• Leukocytes	• Leukocytes
• Lymphocytes	• Lymphocytes	• Lymphocytes
• Macrophage	• Macrophage	• Macrophage
• Monocytes	• Monocytes	• Monocytes
• Natural killer cells	• Natural killer cells	• Natural killer cells
• Neuronal cell	• Neuronal cell	• Neuronal cell
• Neurones	• Neurones	• Neurones
• Neutrophils	• Neutrophils	• Neutrophils
• Oocyte	• Oocyte	• Oocyte
• Pericytes	• Pericytes	• Pericytes
• Platelet	• Platelet	• Platelet
• Smooth muscle cells	• Smooth muscle cells	• Smooth muscle cells
• T cells	• T cells	• T cells
		• B cell
• Tissue chunk	• Tissue chunk	• Tissue chunk
• Tissue section (thin)	• Tissue section (thin)	• Tissue section (thin)

• Tissue slice	• Tissue slice	• Tissue slice
• Whole organ	• Whole organ	• Whole organ
	**Oncogenes**	
	• cKIT-D816	
	• KRAS-G12D	
	• FLT3-ITD	
	• NPM ALK	
	• MLL-AF9	
	• IDH1	
	• VHL	
	• ATF6	
	• BRAF-V600E	
	• CTNNB1	
	• PTEN loss / KMT9A	
	• BAP1	
	• KRAS	
	• KMT2A	
	• AKT1	
	• cMyc	
	• NFE2	
**Sample processing**	**Sample Processing**	**Sample processing**
• Cleared fixed tissue	• Cleared fixed tissue	• Cleared fixed tissue
• Formaldehyde-fixed and paraffin-embedded (FFPE)	• Formaldehyde-fixed and paraffin-embedded (FFPE)	• Formaldehyde fixed and parafin embedded (FFPE)
• Formaldehyde fixation	• Formaldehyde fixation	• Formaldehyde-fixation
• High-pressure frozen	• High-pressure frozen	• High pressure frozen
• None (Physiological solution)	• None (Physiological solution)	• None (Physiological solution)
• OCT embedded and frozen	• OCT embedded and frozen	• OCT embedded and frozen
**Readout**	**Readout**	**Readout**
**Biomechanics**	**Biomechanics**	**Biomechanics**
• Cell stretching	• Cell stretching	• Cell stretching
• Nanoindentation	• Nanoindentation	• Nanoindentation
• Sarcomer Length	• Sarcomer Length	• Sarcomer Length
• Single cell stretching	• Single cell stretching	• Single cell stretching
• Tissue stretching	• Tissue stretching	• Tissue stretching
• Tissue stretching Myodish	• Tissue stretching Myodish	• Tissue stretching Myodish
**Clinical Imaging**	**Clinical Imaging**	**Clinical Imaging**
• Computed tomography scan	• Computed tomography scan	• Computed tomography scan
• Echocardiography	• Echocardiography	• Echocardiography
• Magnetic resonance imaging	• Magnetic resonance imaging	• Magnetic resonance imaging
• Positron emission tomography	• Positron emission tomography	• Positron emission tomography

• Computational modeling	• Computational modeling	**Computational modelling**
		• Quantitative Trait Locus (QTL)
		• Differential Gene Expression (DGE)
		• Cell type analysis
• Electron microscopy	• Electron microscopy	• Electron microscopy
**Electrophysiology (clinical)**	**Electrophysiology (clinical)**	
• Patch Clamp - current clamp	• Patch Clamp - current clamp	
• Patch Clamp - voltage clamp	• Patch Clamp - voltage clamp	
• Sharp Electrode	• Sharp Electrode	
• Electrophysiology (lab)	• Electrophysiology (lab)	
• Flow cytometry / FACS	• Flow cytometry / FACS	**Flow cytometry / FACS**
		**Cytometry**
		• Fluorescence
		• Confocal
**Light microscopy**	**Light microscopy**	**Light microscopy**
• Confocal	• Confocal	• Confocal
• Multiphoton	• Multiphoton	• Multiphoton
• Slidescanner	• Slidescanner	• Slidescanner
• Widefield	• Widefield	• Widefield
		• Fluorescence
**Molecular Biology**	**Molecular Biology**	
• Aptamer (Proteomics)	• Aptamer (Proteomics)	
• Bulk chromatin accessibility	• Bulk chromatin accessibility	
• DNA Methylation (array)	• DNA Methylation (array)	
• Enzyme-linked immunosorbent assay (ELISA)	• Enzyme-linked immunosorbent assay (ELISA)	
• Gene Expression (array)	• Gene Expression (array)	
• Genotyping array and imputation	• Genotyping array and imputation	
• Proximity extension assay (PEA)	• Proximity extension assay (PEA)	
• Quantitative polymerase chain reaction (qPCR)	• Quantitative polymerase chain reaction (qPCR)	
• Standard biochemical assay	• Standard biochemical assay	
• Westernblot	• Westernblot	
		**Cellular/molecular biology**
		• Cellular/Molecular biology
		• Cytokine immune profiling
		• Cell subset composition
		• Cell function & cell states
		• Epigenomics
		• Transcriptome
		• Inflammation

		• Metabolomics
• Optical mapping	• Optical mapping	• Optical mapping
• Optoacoustics	• Optoacoustics	• Optoacoustics
**Sequencing**	**Sequencing**	**Sequencing**
• ATAC sequencing	• ATAC sequencing	• ATAC sequencing
• DNA methylation (WGBS)	• DNA methylation (WGBS)	• DNA methylation (WGBS)
• RNA sequencing	• RNA sequencing	• RNA sequencing
• Single Cell chromatin accessibility	• Single Cell chromatin accessibility	• Single Cell chromatin accessibility
• Single cell RNA seq	• Single cell RNA seq	• Single cell RNA seq
• Whole exome sequencing	• Whole exome sequencing	• Whole exome sequencing
• Whole genome sequencing	• Whole genome sequencing	• Whole genome sequencing
		• CITE-seq
**Mass Spectrometry**	**Mass Spectrometry**	
• Interaction proteomics	• Interaction proteomics	
• Metabolomics	• Metabolomics	
• Proteomics	• Proteomics	
**Probes**		
**Antibodies**		
• alpha-Actinin		
• alpha-SMA (Smooth Muscle Actin)		
• Amphiphysin II		
• ATP5B		
• BDNF		
• beta-Actin		
• beta-Tubulin		
• Bin1		
• CACNA2D1		
• Calnexin		
• CaV1.2 (CACNA1C)		
• Caveolin-3		
• CCL2		
• CD107b / Mac-3		
• CD14		
• CD29		
• CD31		
• CD45		
• CD68		
• CD90.1 (Thy-1.1)		
• Collagen-I-A1 (COL1A1)		

• Connexin 40		
• Connexin 43		
• COUP-TF II/NR2F2		
• CXCL1/GRO-alpha/KC/CINC-1		
• DDR2		
• GAPDH		
• GFP (green fluorescent protein)		
• HCN4		
• Histone 3 (PhosphoS10)		
• Integrin-alpha-1		
• Integrin-beta-1		
• KCa1.1/BK		
• Kir1.2		
• Kir2.1		
• KV1.3		
• KV1.5		
• mCherry		
• Muscarinic Acetycholine Receptor 2/CM2		
• Myosin Light Chain 2		
• Neurofilament Medium		
• p53		
• PDGFR-alpha		
• Phospho-SMAD2		
• Piezo1		
• RFP (red fluorescent protein)		
• Ryanodine Receptor		
• S100B		
• Sarcomeric alpha-Actinin		
• SERCA2 ATPase		
• SLA Class II DR		
• Sodium/Calcium Exchanger		
• SOX10		
• SWC3		
• Talin 1		
• TGF-beta-1		
• Troponin I		
• TRP1		
• TurboGFP		
• Tyrosine Hydroxylase		

• Vimentin		
• YAP		
**Reporter / Marker molecules**		
• BeRST 1		
• CellBrite		
• Cellmask		
• CellTracker		
• CNA-35 (collagen probe)		
• DAPI		
• Dextran		
• DI-4-ANBDQPQ		
• Di-4-ANEPPDHQ		
• Di-4-ANEQ(F)PTEA		
• Fluo-4		
• Fura-2		
• Hoechst 33342		
• Memglow		
• Phalloidin		
• Rhod-2		
• Streptavidin		
• Sytox Dead Cell Stain		
• Tubulin Tracker		
• Ulex Europaeus Agglutinin I (UEA I)		
• Wheat germ agglutinin		
**Stain**		
• Hematoxylin and eosin		
• Horse radish peroxidase		
• Masson's Trichrome		
• Sirius Red		
